# Connexin43 overexpression promoted ferroptosis and increased myocardial vulnerability to ischemia-reperfusion injury in type 1 diabetic mice

**DOI:** 10.7150/ijms.95170

**Published:** 2024-09-09

**Authors:** Yuhui Yang, Jiajia Chen, Jiaqi Zhou, Dongcheng Zhou, Anyuan Zhang, Yuxin Jiang, Jiefu Lin, Weiyi Xia, Yin Cai, Ronghui Han, Yan Lu, Danyong Liu, Zhengyuan Xia

**Affiliations:** 1 Department of Anesthesiology, Affiliated Hospital of Guangdong Medical University, Zhanjiang, Guangdong, 524000, China.; 2 Department of Anesthesiology, The Second Affiliated Hospital and Yuying Children's Hospital, Wenzhou Medical University, Wenzhou, 325027, China.; 3 Department of Health Technology and Informatics, the Hong Kong Polytechnic University, Hong Kong, China.; 4 Doctoral Training Platform for Research and Translation, BoShiWan, ZhongXiang City, Hubei, 431900, China.

## Abstract

Enhancement of Connexin43 (Cx43) and ferroptosis are respectively associated with the exacerbation of myocardial ischemia-reperfusion injury (MIRI) in diabetes. Myocardial vulnerability to ischemic insult has been shown to vary during early and later phases of diabetes in experimental settings. Whether or not Connexin43 (Cx43) and ferroptosis interplay during MIRI in diabetes is unknown. We, thus, aimed to investigate whether or not the content of myocardial Cx43 may be attributable to myocardial vulnerability to MIRI at different stages of diabetes and also to explore the potential interplay between Cx43 and ferroptosis in this pathology. Age-matched control and subgroups of Streptozotocin-induced diabetic mice were subjected to MIRI induced by 30 minutes coronary artery occlusion and 2 hours reperfusion respectively at 1, 2 and 5 weeks of diabetes. Rat cardiac H9C2 cells were exposed to high glucose (HG) for 48h in the absence or presence of Cx43 gene knockdown followed by hypoxia/reoxygenation (HR) respectively for 6 and 12 hours. Post-ischemic myocardial infarct size was reduced in 1 and 2 weeks DM mice concomitant with enhanced GPX4 and reduced cardiac Cx43 and ferroptosis as compared to control. By contrast, cardiac GPX4 was significantly reduced while Cx43 increased at DM 5 weeks (D5w) which was correspondent to significant increases in ferroptosis and myocardial infarction. Post-ischemic cardiac function was improved in 1 and 2 weeks but worsened in 5w DM mice as compared with non-diabetic control. GAP19 (Cx43 inhibitor) significantly attenuated ferroptosis and reduced myocardial infarction in D5w mice. Erastin (ferroptosis activator) reversed the cardioprotective effect of GAP19. In vitro, HR significantly reduced cell viability accompanied with reduced GPX4 but elevated Cx43 expression, MDA production and ferroptosis. Cx43 gene knockdown in H9C2 resulted in a significant increase in GPX4, reduction in MDA and ferroptosis, and subsequently reduced post-hypoxic cell viability. The beneficial effects of Cx43 gene knock-down was minified or eliminated by Erastin. It is concluded that Cx43 overexpression exacerbates MIRI under diabetic conditions via promoting ferroptosis, while its down-regulation at early state of diabetes is attributable to enhanced myocardial tolerance to MIRI.

## Introduction

Myocardial infarction is one of the most common causes of death worldwide^1^. Although restoration of blood flow through reperfusion is the recommended treatment for myocardial infarction, reperfusion itself can cause further damage, known as myocardial ischemia-reperfusion injury (MIRI)^2^. Clinical studies have shown that diabetic patients with ischemic heart disease are at a higher risk of developing MIRI^3^. Additionally, diabetes increases the susceptibility of the heart to ischemia/reperfusion injury (IRI), resulting in larger infarct size and poorer recovery following acute myocardial infarction^4^, ^5^. However, studies on susceptibility and tolerance to MIRI in Type 1 diabetes (T1D) rodents have been inconsistent. Some studies have shown that early T1D mice (1-3 weeks) may have better recovery of heart function and structure after myocardial ischemia-reperfusion than non-diabetic controls^6^, ^7^. This may be due to the fact that the hearts of early T1D mice have better antioxidant capacity and the ability to regulate inflammatory responses. For example, T1D mice may mitigate MIRI-induced oxidative stress and inflammatory damage by increasing the expression and activity of antioxidant enzymes, as well as by modulating the production of inflammatory mediators. However, the hearts of advanced T1D mice (over 5 weeks) had increased susceptibility to MIRI^8^. This may be due to changes in cardiac structure and function caused by chronic hyperglycemia, including cardiomyocyte apoptosis, fibrosis, and disturbance of cardiomyocyte energy metabolism. The susceptibility to this damage may be related to the abnormal distribution or expression of connexin (Cx) 43 (Cx43) in cardiomyocytes caused by diabetes^9^.

Gap junctions are composed of Cx, and six Cx monomers on the plasma membrane form linkers or half-channels, which are intercellular junctions that are widely present in various cell types within organs^9^. The Cx protein family contains nearly 20 subtypes^10^. In the heart, gap junctions are primarily composed of Cx43^9^. Structural and functional changes in Cx43 can regulate the permeability and selectivity of gap junctions, thereby affecting intercellular substance exchange and signal transmission, leading to cellular dysfunction^10^. Cx43 is also involved in channel-independent functions, including regulating cell growth, cell apoptosis, cell cycle, gene transcription, mitochondrial homeostasis, autophagy, and more^11^. Recent study has shown that downregulation of Cx43 can alleviate cisplatin-induced acute kidney injury by inhibiting ferroptosis^12^. However, the mechanism of the interaction between Cx43 and ferroptosis is unclear, especially in the context of MIRI in diabetes.

Ferroptosis, a novel form of programmed cell death, has been identified as being associated with diabetes-related MIRI^13^-^16^. Increase in the generation of reactive oxygen species (ROS) and lipid peroxidation are one of the major initiators of ferroptosis^17^. The main characteristics of ferroptosis include depletion of reduced glutathione (GSH), reduction of the core antioxidant enzyme glutathione peroxidase 4 (GPX4), and increased levels of lipid peroxides^17^. Gao et al. found that inhibiting the catabolism of glutamine (an important component of ferroptosis) can reduce MIRI^18^. However, whether or not there may exist a link between the levels of ferroptosis and the extent of cardiac susceptibility to MIRI in diabetes is unclear.

We hypothesized that Cx43 is involved in regulating the extent of cardiac ferroptosis in diabetes and that the extent of cardiac ferroptosis may be proportional to the susceptibility to MIRI at different stages of diabetes. Our hypothesis was tested in *in vivo* model of MIRI in mice with diabetes and *in vitro* in cultured cardiomyocytes subjected to HR under HG condition.

## Materials and methods

### Animal model establishment

Male animals were selected as the research subject in this paper. Gender differences have been reported for diabetes epidemiology in clinical research, whereas type 1 diabetes has a slight predominance in male adults^19^, ^20^. In addition, the estrogen-mediated protection in MIRI of female mice is a significant interference factor^21^, ^22^. Male C57BL/6 mice (8 weeks old) were obtained from the Guangdong Medical Lab Animal Center and housed in the Laboratory Animal Service Center (Guangdong Medical University, Guangdong, China). The study was approved by the Institutional Ethics Committee of Guangdong Medical University. The diabetes model was established by injecting STZ (freshly dissolved in 0.1M citrate buffer, pH 4.5, Solarbio, China) intraperitoneally at the dose of 50 mg/kg per day for 5 consecutive days as previously described^23^, while control mice were injected with equal volume of citrate buffer. Three days after STZ injection, glucose levels were measured using a glucose analyzer (Beckman Instruments, Fullerton, CA, USA) and mice with hyperglycemia (plasma glucose ≥ 16.7 mmol/L (mM)) were considered diabetic and used for the ensuing experiments. Mice were anesthetized by intraperitoneal injection of 2% pentobarbital (10mg/kg) and maintained at 1.5% isoflurane concentration after endotracheal intubation. I/R was achieved by occluding the left anterior descending (LAD) artery for 30 minutes followed by 2h reperfusion as described previously^24^. The sham group received the same surgical procedure without ligation. The mice were randomly divided into the following groups (n=6-8 per group): Normal control (NC), D1w (Diabetic 1 week), D2w (Diabetic 2 week), D5w (Diabetic 5 week), D1w+IR, D2w+IR, D5w+IR, D5w+GAP19+IR, D5W+Gap19+Erastin+IR groups, which were respectively participated in two parts of in vivo studies as presented in the result sections. One week before myocardial I/R, D5w+GAP19+IR group mice were intraperitoneally injected with GAP19 1mg/kg for a week. The D5W+Gap19+Erastin+IR group mice were intraperitoneally injected with Erastin 20 mg/kg before inducing myocardial IR^25^.

### Echocardiographic assessment

Mouse echocardiograms were monitored using VisualSonics Vevo 3100 imaging system from Fujiflim, Japan. Mice with left chest wall hair removal were placed into a pre-anesthesia box and anesthesia induction was subsequently performed with the volatile anesthetic isoflurane. Isoflurane concentration was set at 4% and oxygen flow rate was 0.5L/min. Subsequently, the mice were fixed in a supine position to the ultrasonic operating table and anesthetized with a nose cone. The operating table temperature was maintained at 37℃. The operating table was rotated to the level of the lower part of the mouse head and foot, and an appropriate amount of ultrasonic coupling agent was applied to the left chest wall. Isoflurane concentration was adjusted to 1% to maintain anesthesia, and the heart rate of each group of mice was controlled between 400 and 450bpm. The ultrasound probe was placed on the short-axis section of the exposed mouse heart. After turning on the M mode, yellow scale was adjusted to the maximum diameter of the heart cavity, and multiple 10s images were collected for evaluating ejection fraction (EF).

### Assessment of myocardial infarct size

The area of myocardial infarction was determined by double staining with Evans-Blue (Solarbio, China) and 2,3,5-triphenyl tetrazolium chloride (TTC, Solarbio, China). The blue-stained area was considered a non-risk area, the unstained area by Evans Blue dye was defined as the area at risk (AAR), and the area not stained by TTC was regarded as the infarcted tissue. The size of myocardial infarction (IS) was calculated as the percentage of infarcted tissue divided by the AAR.

### Biochemical assessment

At the end of reperfusion or reoxygenation, the myocardial tissue, serum samples and H9c2 cells were collected to measure the levels of L-Glutathione (GSH), L-Glutathione Oxidized (GSSG) levels, Total-superoxide dismutase (T-SOD), malonaldehyde (MDA) and labile iron, lactate dehydrogenase (LDH), and creatine kinase (CK)-MB (CK-MB). The levels of GSH, GSSG, T-SOD, MDA (Beyotime Biotechnology, China) and labile iron (BioVision, USA) were measured using assay kits according to the manufacturer's instructions, and the levels of LDH and CK-MB (Donglin Biotechnology, China) were measured by automatic biochemical instruments (HITACHI, Japan).

### Cell culture

H9c2 cells were purchased from the Center for Excellence in Molecular Cell Science (Shanghai, China). The growth medium for the cells consisted of Dulbecco's modified eagle medium (DMEM), 10% fetal bovine serum (FBS) and 100 U/ml penicillin and 100mg/ml streptomycin (Gibco, USA). The cells were divided into four groups: Normal control (NC) group, HG group, HG+HR group, HG+siCx43+HR group, HG+siCx43+Erastin+HR group. Cells were cultured in normal glucose (5.5mM) medium to approximately 50% density and then transferred into high glucose (HG, 35mM) medium. After being exposed to HG for 24h, cells were subjected to hypoxia using a hypoxic incubator (37°C, 94% N2, 5% CO2 and 1% O2) with culture medium deprived of glucose and serum as previously described. After 6h of hypoxia, cells were reoxygenated for 12 h in hyperglycemia medium.

### Cell transfection of siRNA

To block the expression of Cx43, H9c2 cells were added with Cx43 specific small interfering RNAs (siCx43), which were designed and provided by GenePharma as well as their peculiar control. In accordance with the manufacturer's instructions, cell transfection was proceeded with Lipofectamine 3000 (Invitrogen, USA). Six hours (h) later, the previous medium was changed and carried out for further procedures. Silencing of Cx43 were confirmed by western blot or RT-PCR.

### Cell Counting Kit-8 assay

Cells were inoculated in a 96-well plate (8x10³ cells/well) and cultured following the above treatment. Cell viability was assessed by the CCK-8 kit (Dojindo, Japan). Then, 10 µl of CCK-8 solution was added to each well and incubated for 1h at 37°C. The absorbance was measured at 450 nm with a micro plane reader. The cells culture supernatant was taken to a 96-well plate, and then fully mixed with lactate dehydrogenase (LDH) reaction buffer at room temperature according to the procedures of the relevant manufactures (Roche, Switzerland). When the reaction was stopped, the absorbance was measured at 450 nm.

### Fe^2+^ fluorescence assay

Cells were collected and washed with serum-free medium for 3 times. FerroOrange (1μmol/l) was added to the cells for 30 min. Thereafter, the fluorescence intensity of each sample was detected using a multifunctional enzyme labeler (Biotek, USA).

### Flow cytometry

The intracellular ROS levels were measured using a 2,7-dichlorodihydrofluorescein diacetate (DCFH-DA). Annexin V-FITC/PI apoptosis detection kit (Beyotime Technology, China) was utilized to test H9C2 cell apoptosis. Cells were gathered in line with the experimental groups and analyzed according to the manufacturer's description. The ROS level and apoptotic index was detected by flow cytometry.

### Western blotting

Western blot analysis was performed as described previously^26^ using antibodies against Cx43(Proteintech, China), Tubulin (Cell Signaling Technology, USA), GPX4 (Proteintech, China) at 4°C for 12-16 hours then the band intensity was quantified and analyzed by ImageJ.

### Statistical analysis

Statistical analyses were performed using GraphPad Prism (10.1.2). Data were shown as mean ± standard deviation (S.D.). The sample size for each experiment and the replicate number of experiments are reported in the text, Figures or Figure Legends. Continuous variables are presented as mean ± standard deviation (mean ± SD) and assumed to follow a normal distribution. Student's t-test (Student-t) was used for comparisons between two groups, while one-way analysis of variance (One-way ANOVA) was used for comparisons among multiple groups, followed by Tukey's post-hoc test if data were normally distributed and variances were homogeneous. If data did not follow a normal distribution, comparisons between two groups were performed using the Wilcoxon rank-sum test, and comparisons among multiple groups were performed using the Kruskal-Wallis rank-sum test, with Bonferroni correction for pairwise comparisons. P values of 0.05 or less were considered to indicate a statistically significance.

## Results

### Basic characteristics of diabetic mice at the end of experiment

As shown in Table 1, the basic characteristics of STZ-treated mice showed obvious symptoms of diabetes, such as increased blood glucose, water intake, and weight loss, compared with the NC group.

### Early type I diabetic mice were tolerant to MIRI

**Figure 1:**
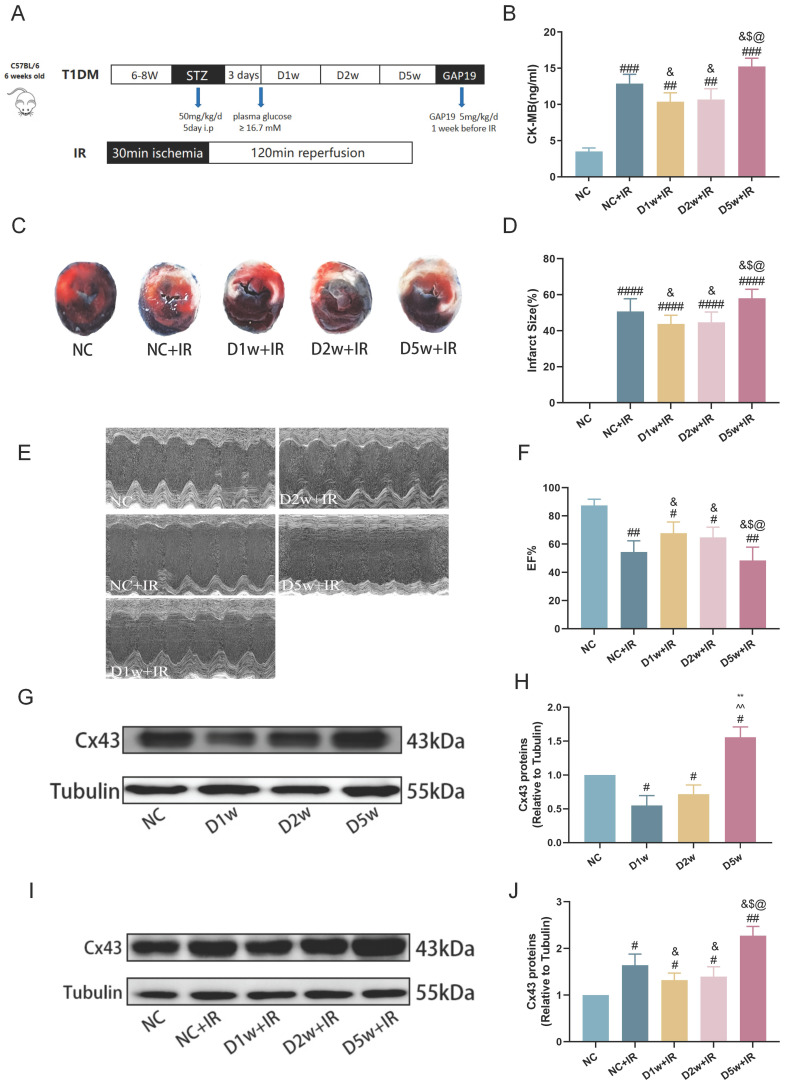
** Early type I diabetic mice were tolerant to MIRI. (A)** 6w mice were injected with STZ at 50 mg/kg/d for 5 consecutive days to induce T1DM. Ischemia reperfusion (I/R) was achieved by 30-minute ischemia followed by 2-hour reperfusion. **(B)** Levels of serum CK-MB, measured after reperfusion using the CK-MB ELISA kit. **(C)** Infarct size (IS) expressed as a percentage of the area at risk (AAR). **(D)** Quantitative data for infarct size. (E) Representative cardiac ultrasound image after IR. (F) EF. (G) Expression of Cx43 in mice with type I diabetes at different stages of diabetes. (H) Expression and quantification of Cx43. Quantification of western blots was performed using Image J. (I) Myocardial Cx43 protein expression after I/R. (J) Quantification of the expression of Cx43. Data are shown as the mean ± SD, n = 6 per group. ^#^*p* < 0.05 vs NC group, ^##^*p* < 0.01 vs NC group, ^###^*p* < 0.001 vs NC group, ^####^*p* < 0.0001 vs NC group, ^&^*p* < 0.05 vs NC+IR group, ^$^*p* < 0.05 vs D1w+IR group, ^$$^*p* < 0.01 vs D1w+IR group, ^@^*p* < 0.05 vs D2w+IR group, ^@@^*p* < 0.01 vs D2w+IR group, ^**^p < 0.01 vs D1w group, ^^^^*p* < 0.01 vs D2w group.

The previous study has reported the cardioprotective effects of acute diabetes^6^. In order to confirm the protective role of acute diabetes and gain a clearer understanding of its underlying mechanisms, we investigated the impact of the duration of diabetes on myocardial tolerance to IRI in STZ-induced diabetes at 1, 2, and 5 weeks after diabetes induction. At early phases of diabetes (i.e., diabetes at 1 week or 2 weeks), the post-ischemic cardiac damage was significantly milder, characterized by smaller myocardial infarct size (Fig. 1C,D), better cardiac function (Fig. 1E,F), and lower plasma levels of CK-MB (Fig. 1B) as compared to that in NC group mice (all p<0.05, D1w+IR or D2w + IR vs. NC + IR). However, in contrast to the D2w and D1w groups, mice at D5w exhibited the largest area of cardiac infarction (Fig. 1C, D), poorer cardiac function (Fig. 1E, F), higher plasma levels of CK-MB (Fig. 1B). At baseline, the expression of Cx43 was significantly decreased in the D1w and D2w groups compared to the NC group, while the cardiac Cx43 in the D5w group of mice was significantly increased as compared to either the control group or to the D1w and D2w groups (Fig. 1G, H). Cx43 in the NC+IR group was significantly increased as compared to NC group (Fig. 1J). It is worth noting that after I/R, levels of cardiac Cx43 expression in D1w and D2w groups were significantly lower than that in the NC+IR group, while Cx43 expression in D5w group was significantly higher than both the NC+IR group or the D1w and D2w groups (Fig. 1I, J). These findings indicated that the heart is protected during the first and second weeks of diabetes, resulting in attenuated IRI, but the damage is exacerbated when facing IR at the 5th week.

### Decreased ferroptosis was associated with enhanced tolerance to MIRI in early type I diabetic mice

**Figure 2:**
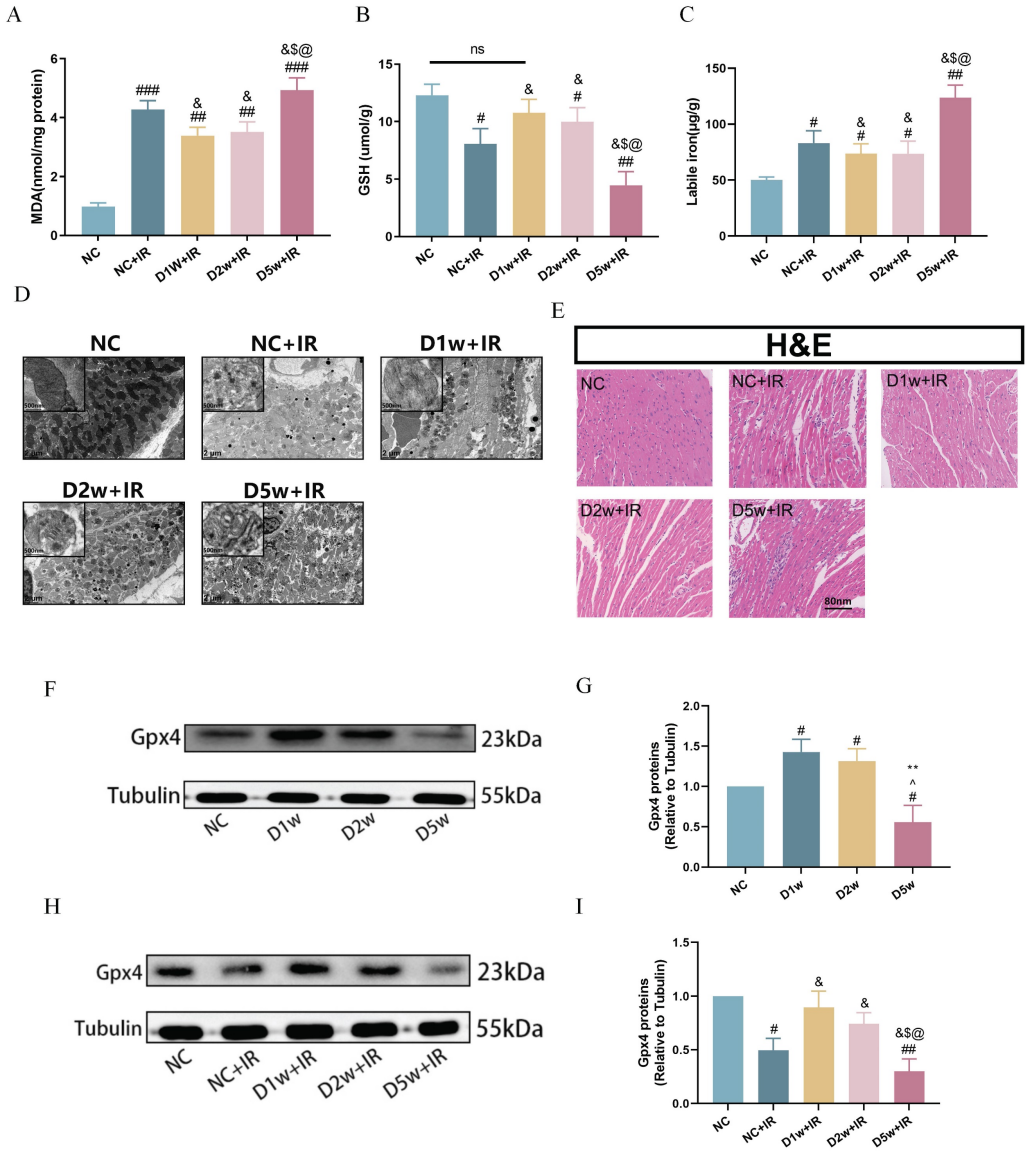
** Decreased ferroptosis was associated with enhanced tolerance to MIRI in early type I diabetic mice.** (A) Cardiac levels of MDA. (B) Alterations of GSH. (C) Labile iron levels. (D)Typical morphological changes of Ferroptosis in cardiomyocytes were observed using transmission electron microscopy (2 μm). (E) Histopathological pictures of heart tissue sections were stained with HE (80 nm). (F) Myocardial GPX4 protein expression in mice with type I diabetes at different periods. (G) Expression of GPX4, Quantification of western blots was performed using Image J. (H) Myocardial GPX4 protein expression after I/R. (I) Quantification of GPX4 protein expression. Data are shown as the mean ± SD, n = 6 per group. ^#^*p* < 0.05 vs NC group, ^##^*p* < 0.01 vs NC group, ^###^*p* < 0.001 vs NC group, ^&^*p* < 0.05 vs NC+IR group, ^$^*p* < 0.05 vs D1w+IR group, ^**^p < 0.01 vs D1w group, ^^^*p* < 0.05 vs D2w group, ^@^*p* < 0.05 vs D2w+IR group, ns means no significance.

Existing study has shown an association between ferroptosis and diabetes-associated MIRI^6^. In this study, we investigated the extent of ferroptosis after ischemia/reperfusion (IR) in mice at 1, 2, and 5 weeks after intraperitoneal injection of STZ. As shown in Figure 2, both normal control and diabetic mice exhibited ferroptosis-related damage after IR.

It is worth noting that the degree of post-ischemic ferroptosis was significantly attenuated in the D1w+IR and D2w +IR groups as compared to that in the NC+IR group, as evidenced by decreased levels of plasma MDA (Fig. 2A) and labile iron (Fig. 2C), along with increased levels of GSH (Fig. 2B), which were consistent with the levels of injury observed after IR. Transmission electron microscopy images also revealed varying degrees of mitochondrial swelling, blurred cristae, and vacuolization in all groups of mice following IR, but the damage was less pronounced in the D1w and D2w groups compared to the NC group, while more severe in the D5w group (Fig. 2D). H&E staining of myocardial sections revealed that after IR, non-diabetic mice exhibited a reduction in myocardial cells, enlarged nuclei, and increased intercellular spaces, while the diabetic groups at 1 and 2 weeks showed lower levels of myocardial cell injury compared to the NC group (Fig. 2E). GPX4 is one of the core enzymes of the antioxidant system (GSH system). We found that at baseline, GPX4 expression was elevated in the D1w and D2w groups compared to the non-diabetic control group, while the trend was completely reversed in the D5w group (Fig. 2F, G). Compared with non-diabetic mice, the expression of GPX4 was up regulated in D1w and D2w groups and decreased in D5w group after I/R (Fig. 2H, I). These findings suggest that the enhanced tolerance of the heart to MIRI during the first and second weeks of diabetes may be attributable to the alleviation of ferroptosis.

### Protective effect of Cx43 inhibitor GAP19 on MIRI in diabetic mice

**Figure 3:**
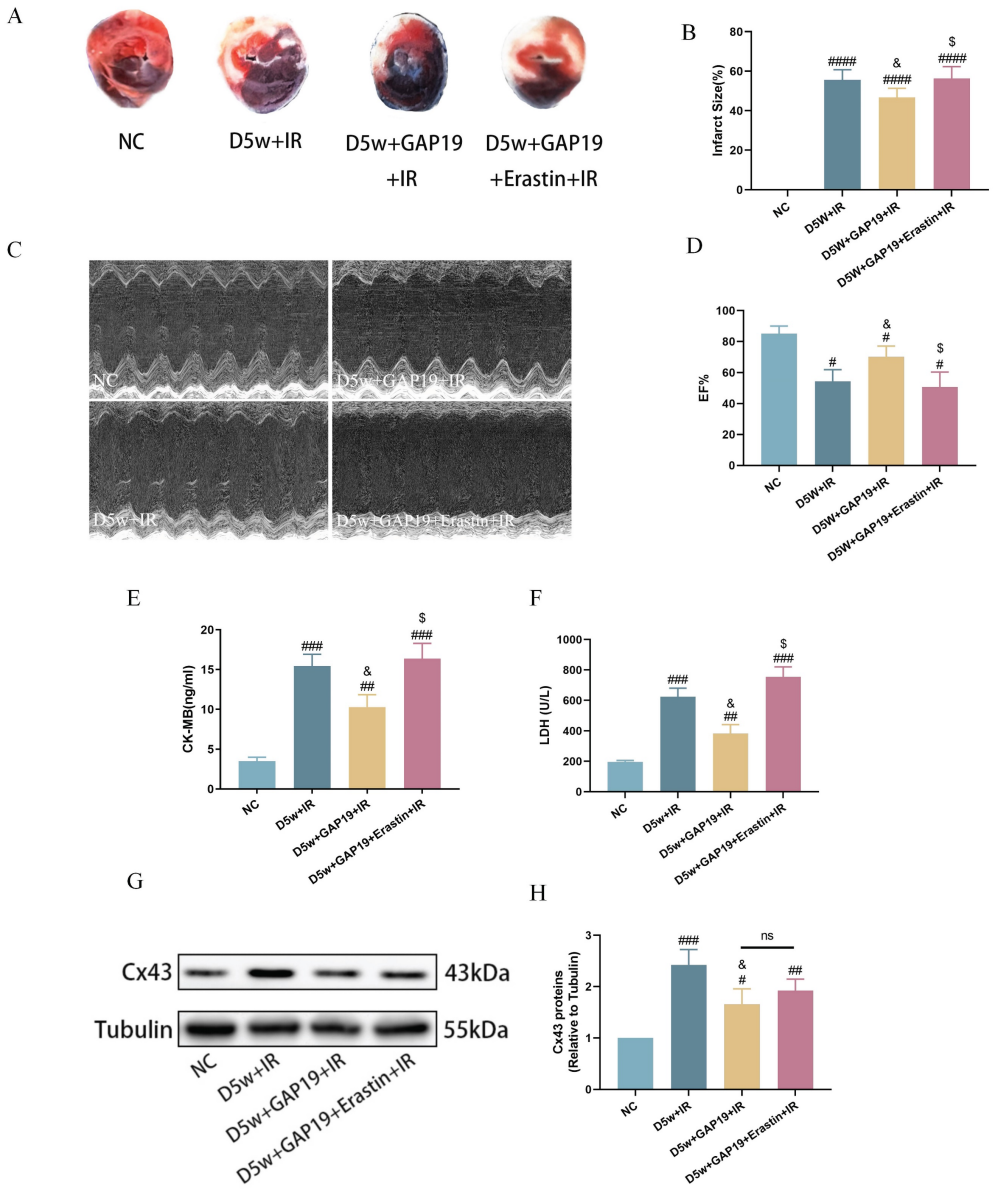
** Effect of Cx43 inhibition on MIRI in diabetic mice.** (A) Representative images for infarct size were detected using TTC and evens blue staining. (B) Quantitative data for infarct size. (C) Cardiac ultrasound and changes of EF after IR. (D) EF. (E) Concentrations of CK-MB. (F) Leakage of LDH. (G) Representative images of protein expression. (H) Quantification of Cx43 protein expression. Data are shown as the mean ± SD, n = 6 per group. ^#^*p* < 0.05 vs NC group, ^##^*p* < 0.01 vs NC group, ^###^*p* < 0.001 vs NC group, ^####^*p* < 0.0001 vs NC group, ^&^*p* < 0.05 vs D5w+IR group, ^$^*p* < 0.05 vs D5w+GAP19+IR group, ns means no significance.

To further investigate the potential interaction between Cx43 and cardiac ferroptosis in MIRI under diabetic conditions, we administered the Cx43 inhibitor GAP19 via continuous intraperitoneal injection in mice for one week. Erastin is an established iron apoptosis inducer that specifically inhibits GPX4 activity, promoting ferroptotic cell death. Bai, et al.'s study^27^ shows that Erastin causes significant ferroptosis in H9C2 at concentrations vary from 2.5 to 10 uM. Our preliminary study also revealed that Erastin could cause significant ferroptosis in H9C2 cells at the concentration of 5 uM (data not shown), thus we used Erastin at 5uM in our ensuring studies with the aim to see if Erastin could partially revert the effects of Cx43 inhibition with GAP19. As shown in Figure 3, compared to the D5w+IR group, the D5w+GAP19+IR group exhibited milder damage, characterized by a smaller myocardial infarct size (Fig. 3A, B), higher left ventricular EF (Fig. 3C, D), and lower plasma levels of CK-MB (Fig. 3E) and LDH (Fig. 3F). However, in the D5w+GAP19+Erastin+IR group, the damage was aggravated once again (Fig. 3A-F). In addition, compared with the control group, Cx43 expression was significantly increased in the D5w+IR group and significantly decreased after GAP19 treatment, while Erastin had no significant effect on Cx43 expression (Fig. 3G, H) which is indicative that Erastin reversed Cx43 inhibition-mediated protection against MIRI and that inhibition of ferroptosis is a major mechanism whereby GAP19 conferred therapeutic effects against diabetic MIRI.

### Cx43 inhibitor attenuated ferroptosis in MIRI in type I diabetic mice

**Figure 4:**
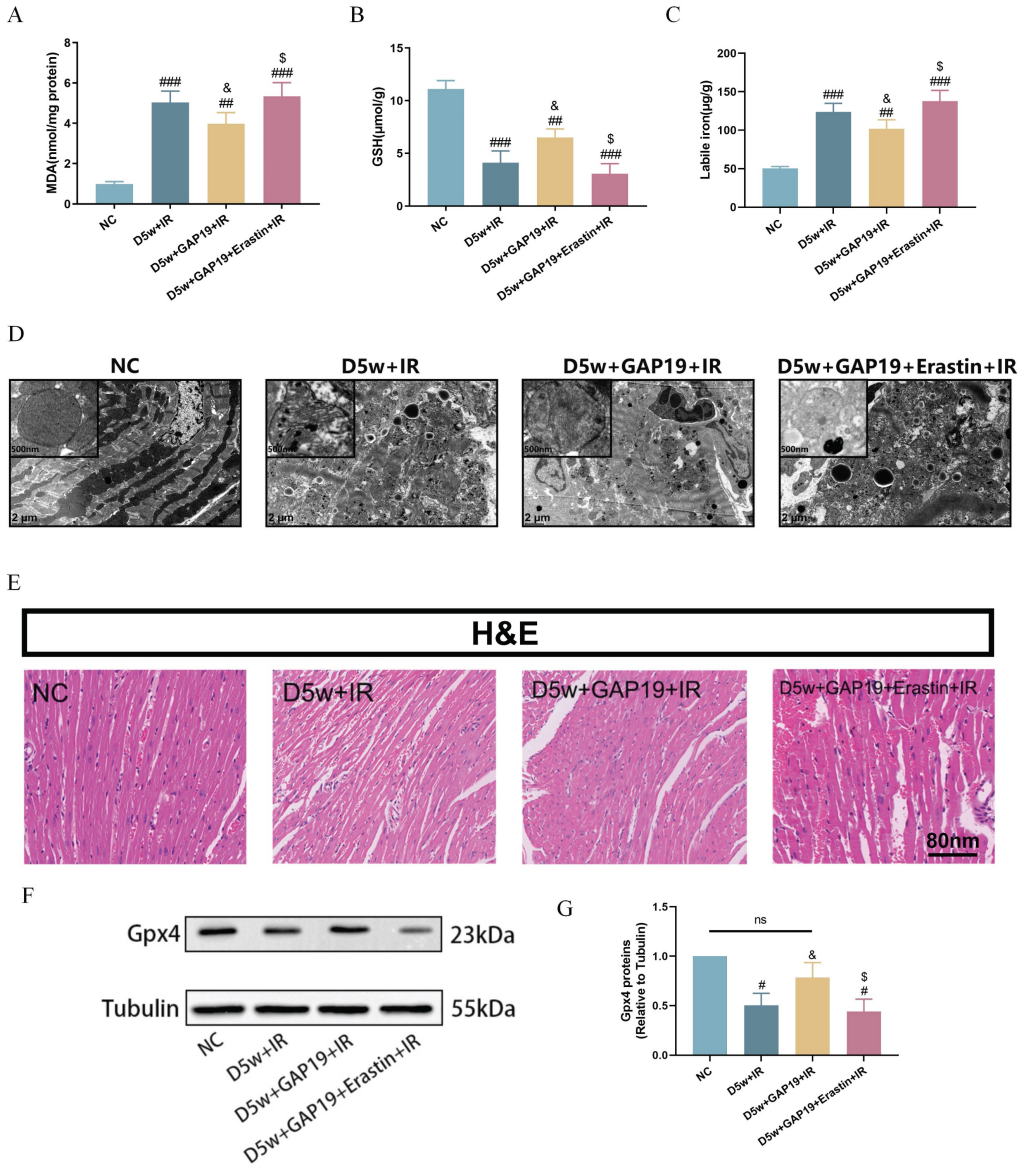
** Cx43 inhibitor GAP19 attenuated ferroptosis in MIRI in type I diabetic mice.** (A) Degree of MDA. (B) Alterations of GSH. (C) Labile iron levels. (D) Transmission electron microscopy (2 μm). (E) Histopathological pictures of heart tissue sections were stained with HE (80 nm). (F) Representative images of protein expression. (G) Quantification of Gpx4 protein expression. Data are shown as the mean ± SD, n = 6 per group. ^#^*p* < 0.05 vs NC group, ^##^*p* < 0.01 vs NC group, ^###^*p* < 0.001 vs NC group, ^&^*p* < 0.05 vs D5w+IR group, ^$^*p* < 0.05 vs D5w+GAP19+IR group, ns means no significance.

As shown in Figure 4, compared to the D5w+IR group, the D5w+GAP19+IR group exhibited significantly attenuated ferroptosis, characterized by higher levels of serum GSH (Fig. 4B) and reduced labile iron (Fig. 4C) and MDA levels (Fig. 4A). Ultrastructural analysis of cardiac tissue revealed that in the D5w+IR group, most of the mitochondrial cristae were completely lost and the mitochondrial membranes were incomplete, whereas in the D5w+GAP19+IR group, these abnormalities were reduced (Fig. 4D). Furthermore, compared to the D5w+GAP19+IR group, the D5w+GAP19+Erastin+IR group showed higher levels of serum MDA (Fig. 4A) and labile iron (Fig. 4C), lower GSH levels (Fig. 4B), and more severe mitochondrial damage in cardiac tissue (Fig. 4D). H&E staining of cardiac tissue sections revealed more severe injury in the D5w+GAP19+Erastin+IR group compared to the D5w+GAP19+IR group (Fig. 4E). In addition, compared with the control group, GPX4 expression was significantly decreased in the D5w+IR group, and significantly increased after GAP19 treatment, while GPX4 expression was again suppressed in the D5w+IR+GAP19+Erastin group (Fig. 4F, G). These results suggest that GAP19 mitigates MIRI in diabetic mice by inhibiting ferroptosis.

### High glucose exacerbated hypoxia-reoxygenation induced H9C2 cardiomyocyte ferroptosis

**Figure 5:**
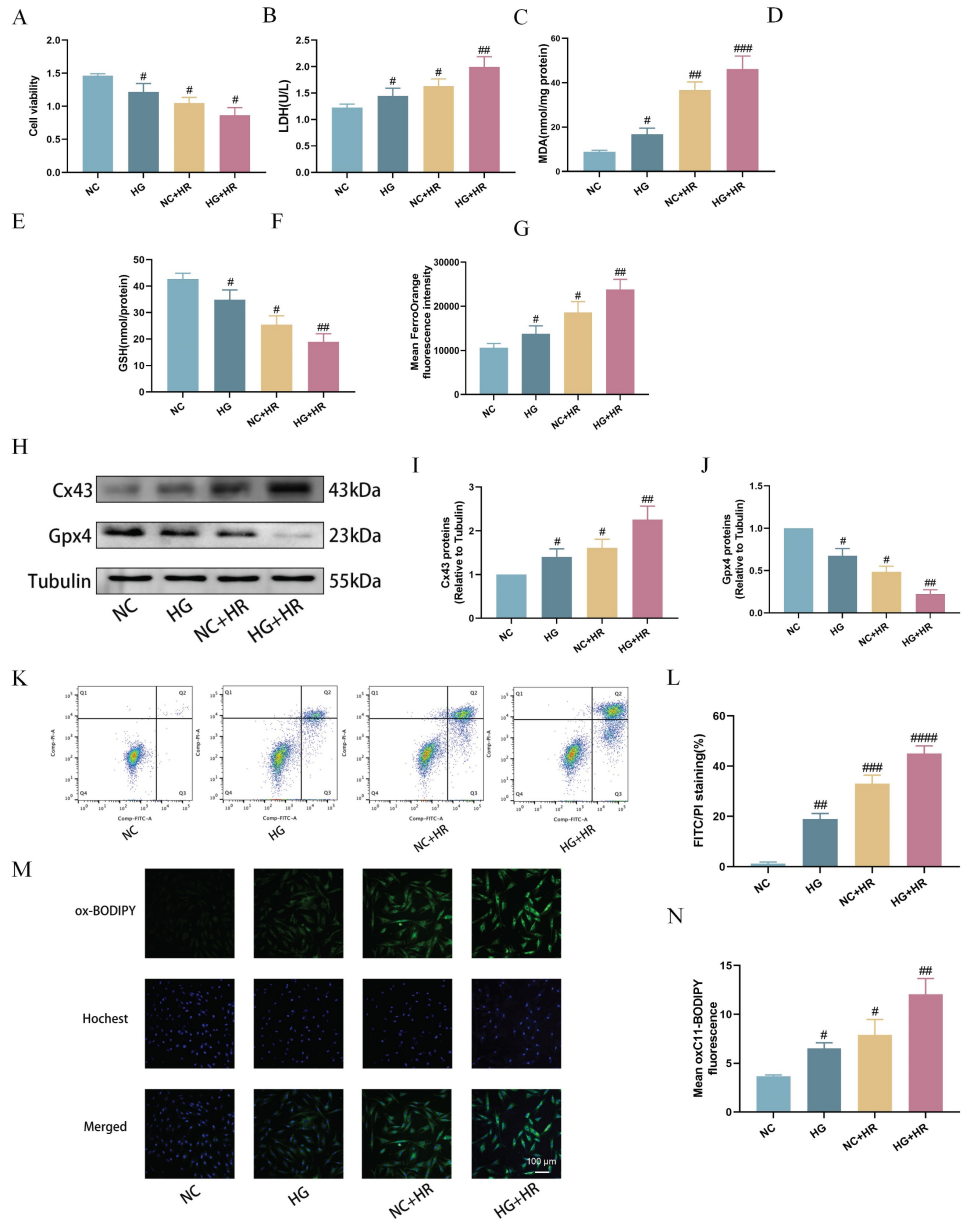
** Expose of H9C2 cardiomyocytes to hypoxia-reoxygenation (HR) under high glucose (HG) led to increased ferroptosis.** (A) Cell viability. (B) Leakage of LDH. (C) Content of MDA. (D) and (E) Alterations of GSH and GSH/GSSG levels. (F) Level of T-SOD. (G) FerroOrange fluorescence intensity. (H) Representative images of Cx43 and Gpx4 protein expression. (I) Quantification of Cx43 protein expression. (J) Quantification of Gpx4 protein expression. (K) Counts of apoptotic cells by flow cytometry. (L) Quantification of apoptotic ratio. (M) lipid peroxidation measured by C11 BODIPY (100 µm). (N) Quantification of C11 BODIPY. Data are shown as the mean ± SD, n = 6 per group. ^#^*p* < 0.05 vs NC group, ^##^*p* < 0.01 vs NC group, ^###^*p* < 0.001 vs NC group, ^####^*p* < 0.0001 vs NC group.

Subsequently, we established an *in vitro* model using H9C2 cardiomyocytes exposed to HG prior to inducing HR injury at the cellular level. As shown in Figure 5, compared to the control group, cell viability was significantly reduced after exposure to HG or HR, and the HG and HR in combination further exacerbated the decrease in cell viability (Fig. 5A). Additionally, compared to NC group, the HG+HR group exhibited significantly elevated levels of LDH (Fig. 5B), MDA (Fig. 5C), Fe2+ levels (Fig. 5G), cell apoptosis (Fig. 5K, L), and lipid peroxidation (Fig. 5M, N), along with decreased levels of GSH (Fig. 5D), GSH/GSSG ratio (Fig. 5E), and T-SOD (Fig. 5F). Furthermore, compared to the control group, Cx43 expression was increased in both the HG and the HR groups, and significantly elevated in the HG+HR group, while the expression of GPX4 was decreased in the HG and HR groups, and significantly decreased in the HG+HR group (Fig. 5H-J). These results indicate that ferroptosis is a major form of cell injury in H9C2 cells exposed to HG and/or HR in the current experimental settings.

### Cx43 gene knock-down attenuated ferroptosis in H9C2 cells exposed to HG/HR

**Figure 6:**
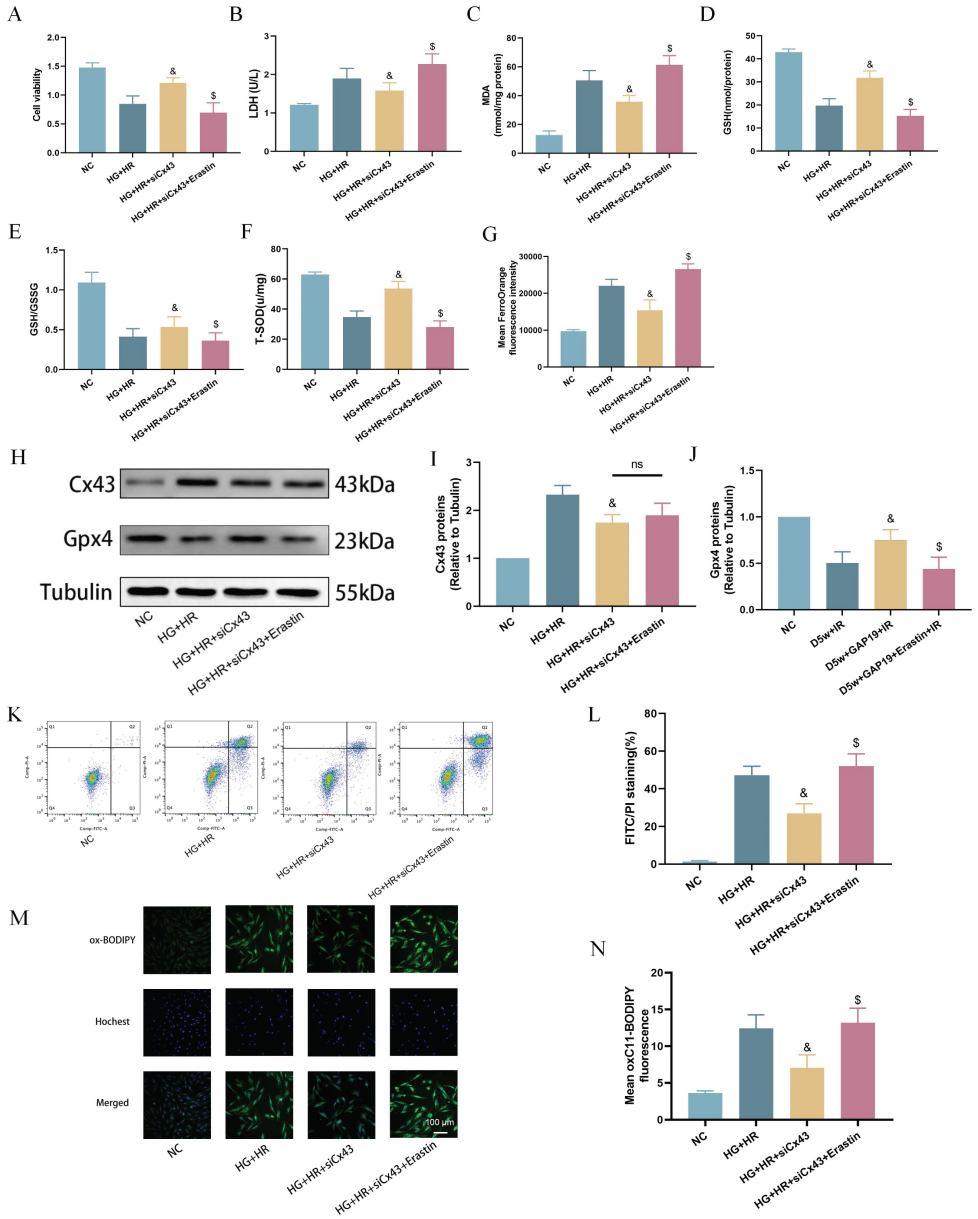
** Downregulation of Cx43 attenuated ferroptosis in H9C2 cells exposed to HG/HR.** (A) Cell viability. (B) Leakage of LDH. (C) Contents of MDA. (D) and (E) Alterations of GSH and GSH/GSSG levels. (F) Level of T-SOD. (G) FerroOrange fluorescence intensity. (H) Representative images of protein expression. (I) Quantification of Cx43 protein expression. (J) Quantification of Gpx4 protein expression. (K) Counts of apoptotic cells assessed by flow cytometry. (L) Quantification of apoptotic ratio. (M) lipid peroxidation measured by C11 BODIPY (100 µm). (N) Quantification of of C11 BODIPY. Data are shown as the mean ± SD, n = 6 per group. ^&^*p* < 0.05 vs HG+HR group, ^$^*p* < 0.05 vs HG+HR+siCx43 group, ns means no significance.

To further validate the potential interaction between Cx43 and ferroptosis in cardiomyocytes under HG/HR, we used specific siRNA to knock down Cx43 in H9C2 cells and performed subsequent experiments. As shown in Figure 6, compared to the HG+HR group, post-hypoxic cell viability was significantly increased after Cx43 knockdown (Fig. 6A). In comparison to the HG+HR+siCx43 group, cell viability was significantly reduced in the HG+HR+siCx43+Erastin group (Fig. 6A), accompanied by significant increases in levels of LDH (Fig. 6B), MDA (Fig. 6C), Fe2+ levels (Fig. 6G), cell apoptosis (Fig. 6K,L), and lipid peroxidation (Fig. 6M,N), as well as significant decreases in levels of GSH (Fig. 6D), GSH/GSSG ratio (Fig. 6E), and T-SOD (Fig. 6F). Protein imprinting experiments revealed that in the HG+HR+siCx43+Erastin group, Cx43 levels did not significantly change, while GPX4 levels were significantly decreased compared to the HG+HR+siCx43 group (Fig. 6H-J). These results indicated that Erastin reversed the protective effect of Cx43 downregulation on HG+HR-induced injury in H9C2 cardiomyocytes.

## Discussion

Our research found that compared to the control group, STZ-induced diabetic mice at 1-2 weeks showed less damage after I/R, along with a decrease in the expression level of Cx43 with concomitant increase in the expression level of GPX4 and reduction in ferroptosis. By contrast, post-ischemic myocardial injury was more severe at 5-week diabetes, and the increased MIRI are associated with increases in cardiac Cx43 and ferroptosis. Subsequently, we used the Cx43 inhibitor GAP19 in 5-week diabetic mice, which reduced ferroptosis and decreased the size of myocardial infarction after IR. The protective effect of GAP19 was reversed when Erastin was applied.

Given the previous findings on the ability of Erastin to induce ferroptosis, we conducted further investigation into its role under our specific experimental conditions. Although we did not have a dedicated experimental group for Erastin alone, the available data sufficiently support our conclusion that ferroptosis induction with Erastin can counteract the protective effects of Cx43 inhibition. Furthermore, in an *in vitro* H9C2 cell model, silencing the expression of Cx43 alleviated ferroptosis induced by high glucose, hypoxia, and reoxygenation. This study is the first to report that the downregulation of Cx43 expression is associated with reduced ferroptosis and enhanced tolerance to MIRI in early-stage type 1 diabetic rodents while increased Cx43 and subsequent increase in ferroptosis at late-stage diabetes (i.e., 5-week diabetes in the current study) are attributable to increased myocardial susceptibility to MIRI in diabetes.

Some previous animal experiments have also indicated that the early-stages diabetic heart exhibits a certain degree of tolerance to IR, and the relationship between ferroptosis and the susceptibility to MIRI in diabetes remains to be explored^6^. In this study, we assessed the vulnerability of mouse myocardium at different stages of diabetes to MIRI. Our experimental results demonstrated that early-stage diabetes (1-2 weeks) enhances the tolerance of mouse myocardium to IR, reducing myocardial infarct size, inhibiting serum levels of CK-MB and LDH, and suppressing inflammation and lipid peroxidation induced by MIRI. During this process, ferroptosis in myocardial tissue is alleviated. In vitro experiments revealed reduced cell viability and significant increased ferroptosis in H9C2 cardiomyocytes subjected to HG+HR, similar to the situation seen in 5-week diabetic mice.

Ferroptosis is a newly discovered iron-dependent programmed cell death mode and is one of the mechanisms underlying diabetes-related MIRI^15^, ^28^. One of the main mechanisms of ferroptosis is the reduction of GPX4, a core regulatory enzyme in the antioxidant system (GSH system)^29^. When lipid peroxides accumulate to a certain extent in cells, the insufficient activity of GPX4 leads to the ineffective clearance of lipid peroxides^29^. Insufficient GPX4 activity increases oxidative stress within cells, further promoting the accumulation of iron ions and the diffusion of lipid peroxidation. This process leads to cell membrane damage, impaired mitochondrial function, and abnormal function of other organelles, ultimately resulting in cell death^30^. Our experimental results demonstrate that the application of GAP19 in IR mice at D5w reduces oxidative stress and myocardial injury, while increasing GPX4 protein expression. Erastin is a small molecule that induces ferroptosis through the xc/GPX4 mechanism. Therefore, Erastin reverses the protective effect of GAP19 on the myocardium. Furthermore, in *in vitro* experiments, we found that Erastin can reverse the protective effects of Cx43 knockdown on HG/HR-induced H9C2 cardiomyocytes.

In addition, ROS are potent oxidative stress signals that can directly damage cells or tissues. Excessive ROS production leads to cellular oxidative damage and is associated with reduced levels of the core regulatory enzyme GPX4^29^, ^31^. During myocardial IR, the endogenous antioxidant capacity of the myocardium is insufficient, resulting in massive ROS production during the ischemic and reperfusion periods, leading to myocardial injury^32^-^34^. Diabetes further increases ROS release, inhibits GPX4, exacerbates IR-induced damage^15^, ^35^. These studies have shown that ROS is one of the main mechanisms of MIRI and is closely related to ferroptosis. Considering the continuous diffusion of ROS mediated by Cx43 channels between adjacent cells, we propose that Cx43-mediated ROS propagation directly damages neighboring cells or activates downstream pathways associated with ferroptosis, which may be one of the most important mechanisms underlying the abnormal susceptibility of diabetic myocardium to MIRI at different stages. In human heart tissue, the gap junction mainly relies on Cx43, which forms a normally closed half-channel^9^. When it is over-opened, it can transmit a "death signal" that amplifies and intensifies cytotoxicity^36^, ^37^. Our study confirmed that inhibition of Cx43-mediated ROS over production can alleviate the damage caused by diabetic myocardial ischemia-reperfusion.

Cx43 is the most widely distributed connexin in the human body, and Cx43 channels have greater permeability to cell signals^9^. These characteristics determine the more significant role of Cx43 in regulating cellular life activities. For example, it has been reported that high blood glucose inhibits the expression of Cx43 in brain cells, which is related to impaired communication between astrocytes^38^. Previous studies have shown variability in the expression of Cx43 in the heart under diabetic conditions, including increased^39^ , unchanged^40^ , and decreased expression^41^, but the mechanism that causes this phenomenon is still unknown. In this study, we examined the expression of Cx43 in streptozotocin-induced diabetes and found a reduction in Cx43 expression in the myocardium of early-stage diabetes (1-2 weeks) mice compared to the control group. The experimental results indicate that inflammation and lipid peroxidation in the myocardial tissue of early-stage diabetic mice are significantly reduced when the myocardium undergoes ischemia-reperfusion (IR). Additionally, the levels of free iron in the myocardium decrease, and mitochondrial damage is reduced. These results suggest that early-stage diabetic mice may enhance the tolerance of myocardial tissue to IR by reducing the ROS propagation between adjacent cells through Cx43 channels and alleviating downstream pathways associated with ferroptosis. However, compared with early-stage diabetic mice (1-2 weeks), Cx43 expression and ROS production were significantly increased in myocardial tissue of 5-week diabetic mice, and the damage caused by ferroptosis was more pronounced.

Additionally, in the early stage of type 1 and type 2 diabetes, the protective effect on myocardial ischemia-reperfusion injury has been observed in several studies, the underlying main mechanism involves a number of factors. Oxidative stress is commonly regarded as one of the important contributing factors in the pathogenesis of diabetes mellitus. Generally, oxidative stress is the result of an excessive production ROS, which could be counteracted by endogenous antioxidants under normal physiological conditions. Our team's previous study found that at the first week of diabetes, some of endogenous antioxidants (such as SOD) complementarily increased^42^, which is also in line with the clinical situation in early stages of type 1 diabetes in children^43^. Studies also indicate that the nitric oxide (NO) synthase (NOS) activity and NO levels were increased in diabetic rats mainly after 14 days of streptozotocin induced diabetes, providing the protective effect to the overall cardiovascular system^44^, ^45^. Besides, early diabetes affected the cardiac membrane phospholipid fatty acid composition by increasing the arachidonic acid and n-3 polyunsaturated fatty acids levels, which may help the heart to resist some sudden cardiac damage at this early phase of diabetes^46^. We attribute these to the consequence of body's own stress protection effect exerted by hyperglycemia, leading to the seen phenomenon of increased tolerance against ischemia-reperfusion injury during the first 2 weeks of streptozotocin-induced diabetes. Additional, metabolism shift between early and late stage in streptozotocin-induced diabetes has been found in rat kidney mitochondria^47^. Given that Cx43 also plays a key role in cardiac mitochondrial function^48^, we believe that similar or even more complicated metabolic response exist during different stage of MIRI in subjects with diabetes. Knowing the metabolic process may be the next key point to better understand and apply treatment to diabetic patients who facing ischemic cardiac injury.

## Conclusion

In the early stages of diabetic myocardium, the downregulation of Cx43 inhibits ferroptosis, thereby enhancing myocardial tolerance to MIRI. However, in the later stages of diabetes, the increased level of Cx43 gradually diminishes this tolerance, eventually lead to reduced tolerance to MIRI. Cx43 may be a potential therapeutic target for clinical treatment of myocardial ischemia-reperfusion injury in patients with diabetes.

## Figures and Tables

**Table 1:** Basic characteristics of diabetic mice at the end of experiment.

Group	Body weight (g)	Food intake (g/d)	Water intake (ml/kg/d)	Blood glucose (mmol/L)
NC	24.79 ± 2.49	6.72 ± 1.84	418.39 ± 30.02	6.29 ± 1.64
D1w	23.34 ± 1.75	8.66 ± 2.07*	677.28 ± 95.46*	25.54 ± 4.75**
D2w	21.57 ± 2.62*	8.53 ± 1.54*	726.17 ± 106.65*	24.72 ± 5.28**
D5w	20.23 ± 2.74*	9.30 ± 1.78*	739.97 ± 186.41*	24.19 ± 6.86**

Data are shown as mean ± SD. n = 15 per group, water intake and food consumption values were the average value of corresponding weeks. Body weight and plasma glucose were measured on the day of execution or surgery. ^*^p < 0.05 vs NC group, ^**^p < 0.01 vs NC group.

## References

[1] Li S, Lear SA, Rangarajan S, Hu B, Yin L, Bangdiwala SI (2022). Association of Sitting Time With Mortality and Cardiovascular Events in High-Income, Middle-Income, and Low-Income Countries. JAMA Cardiol.

[2] Lefer DJ, Marbán E (2017). Is Cardioprotection Dead?. Circulation.

[3] Glovaci D, Fan W, Wong ND (2019). Epidemiology of Diabetes Mellitus and Cardiovascular Disease. Curr Cardiol Rep.

[4] Rezende PC, Rahmi RM, Hueb W (2016). The Influence of Diabetes Mellitus in Myocardial Ischemic Preconditioning. J Diabetes Res.

[5] Ng ACT, Delgado V, Borlaug BA, Bax JJ (2021). Diabesity: the combined burden of obesity and diabetes on heart disease and the role of imaging. Nat Rev Cardiol.

[6] Ma G, Al-Shabrawey M, Johnson JA, Datar R, Tawfik HE, Guo D (2006). Protection against myocardial ischemia/reperfusion injury by short-term diabetes: enhancement of VEGF formation, capillary density, and activation of cell survival signaling. Naunyn Schmiedebergs Arch Pharmacol.

[7] Balakumar P, Sharma NK (2012). Healing the diabetic heart: does myocardial preconditioning work?. Cell Signal.

[8] Fang B, Liu F, Yu X, Luo J, Zhang X, Zhang T (2023). Liraglutide alleviates myocardial ischemia-reperfusion injury in diabetic mice. Mol Cell Endocrinol.

[9] Schulz R, Görge PM, Görbe A, Ferdinandy P, Lampe PD, Leybaert L (2015). Connexin 43 is an emerging therapeutic target in ischemia/reperfusion injury, cardioprotection and neuroprotection. Pharmacol Ther.

[10] Beyer EC, Berthoud VM (2018). Gap junction gene and protein families: Connexins, innexins, and pannexins. Biochim Biophys Acta Biomembr.

[11] Van Campenhout R, Cooreman A, Leroy K, Rusiecka OM, Van Brantegem P, Annaert P (2020). Non-canonical roles of connexins. Prog Biophys Mol Biol.

[12] Yu M, Lin Z, Tian X, Chen S, Liang X, Qin M (2021). Downregulation of Cx43 reduces cisplatin-induced acute renal injury by inhibiting ferroptosis. Food Chem Toxicol.

[13] Yu Q, Zhang N, Gan X, Chen L, Wang R, Liang R (2023). EGCG attenuated acute myocardial infarction by inhibiting ferroptosis via miR-450b-5p/ACSL4 axis. Phytomedicine.

[14] Huang Q, Tian L, Zhang Y, Qiu Z, Lei S, Xia ZY (2023). Nobiletin alleviates myocardial ischemia-reperfusion injury via ferroptosis in rats with type-2 diabetes mellitus. Biomed Pharmacother.

[15] Li W, Li W, Leng Y, Xiong Y, Xia Z (2020). Ferroptosis Is Involved in Diabetes Myocardial Ischemia/Reperfusion Injury Through Endoplasmic Reticulum Stress. DNA Cell Biol.

[16] Li W, Li W, Wang Y, Leng Y, Xia Z (2021). Inhibition of DNMT-1 alleviates ferroptosis through NCOA4 mediated ferritinophagy during diabetes myocardial ischemia/reperfusion injury. Cell Death Discov.

[17] Chen X, Li J, Kang R, Klionsky DJ, Tang D (2021). Ferroptosis: machinery and regulation. Autophagy.

[18] Gao M, Monian P, Quadri N, Ramasamy R, Jiang X (2015). Glutaminolysis and Transferrin Regulate Ferroptosis. Mol Cell.

[19] Wändell PE, Carlsson AC (2013). Time trends and gender differences in incidence and prevalence of type 1 diabetes in Sweden. Curr Diabetes Rev.

[21] Booth EA, Lucchesi BR (2008). Estrogen-mediated protection in myocardial ischemia-reperfusion injury. Cardiovasc Toxicol.

[22] Chen J, Liu Y, Pan D, Xu T, Luo Y, Wu W (2022). Estrogen inhibits endoplasmic reticulum stress and ameliorates myocardial ischemia/reperfusion injury in rats by upregulating SERCA2a. Cell Commun Signal.

[23] Chen W, Wang X, Sun Q, Zhang Y, Liu J, Hu T (2022). The upregulation of NLRP3 inflammasome in dorsal root ganglion by ten-eleven translocation methylcytosine dioxygenase 2 (TET2) contributed to diabetic neuropathic pain in mice. J Neuroinflammation.

[24] Li H, Yao W, Liu Z, Xu A, Huang Y, Ma XL (2016). Hyperglycemia Abrogates Ischemic Postconditioning Cardioprotection by Impairing AdipoR1/Caveolin-3/STAT3 Signaling in Diabetic Rats. Diabetes.

[25] Wang X, Wang Y, Huang D, Shi S, Pei C, Wu Y (2022). Astragaloside IV regulates the ferroptosis signaling pathway via the Nrf2/SLC7A11/GPX4 axis to inhibit PM2.5-mediated lung injury in mice. Int Immunopharmacol.

[26] Zhou J, Xia W, Chen J, Han K, Jiang Y, Zhang A (2024). Propofol and salvianolic acid A synergistically attenuated cardiac ischemia-reperfusion injury in diabetic mice via modulating the CD36/AMPK pathway. Burns Trauma.

[27] Bai YT, Xiao FJ, Wang H, Ge RL, Wang LS (2021). Hypoxia protects H9c2 cells against Ferroptosis through SENP1-mediated protein DeSUMOylation. Int J Med Sci.

[28] He J, Liu D, Zhao L, Zhou D, Rong J, Zhang L (2022). Myocardial ischemia/reperfusion injury: Mechanisms of injury and implications for management (Review). Exp Ther Med.

[29] Park TJ, Park JH, Lee GS, Lee JY, Shin JH, Kim MW (2019). Quantitative proteomic analyses reveal that GPX4 downregulation during myocardial infarction contributes to ferroptosis in cardiomyocytes. Cell Death Dis.

[30] Yu P, Zhang J, Ding Y, Chen D, Sun H, Yuan F (2022). Dexmedetomidine post-conditioning alleviates myocardial ischemia-reperfusion injury in rats by ferroptosis inhibition via SLC7A11/GPX4 axis activation. Hum Cell.

[31] Chouchani ET, Pell VR, Gaude E, Aksentijević D, Sundier SY, Robb EL (2014). Ischaemic accumulation of succinate controls reperfusion injury through mitochondrial ROS. Nature.

[32] Shen S, He F, Cheng C, Xu B, Sheng J (2021). Uric acid aggravates myocardial ischemia-reperfusion injury via ROS/NLRP3 pyroptosis pathway. Biomed Pharmacother.

[33] Wang R, Dong S, Xia R, Sun M, Sun Y, Ren H (2023). Kinsenoside mitigates myocardial ischemia/reperfusion-induced ferroptosis via activation of the Akt/Nrf2/HO-1 pathway. Eur J Pharmacol.

[34] Song R, Dasgupta C, Mulder C, Zhang L (2022). MicroRNA-210 Controls Mitochondrial Metabolism and Protects Heart Function in Myocardial Infarction. Circulation.

[35] Lei S, Su W, Xia ZY, Wang Y, Zhou L, Qiao S (2019). Hyperglycemia-Induced Oxidative Stress Abrogates Remifentanil Preconditioning-Mediated Cardioprotection in Diabetic Rats by Impairing Caveolin-3-Modulated PI3K/Akt and JAK2/STAT3 Signaling. Oxid Med Cell Longev.

[36] Schulz R, Heusch G (2004). Connexin 43 and ischemic preconditioning. Cardiovasc Res.

[37] Theodoric N, Bechberger JF, Naus CC, Sin WC (2012). Role of gap junction protein connexin43 in astrogliosis induced by brain injury. PLoS One.

[38] Garvin J, Semenikhina M, Liu Q, Rarick K, Isaeva E, Levchenko V (2022). Astrocytic responses to high glucose impair barrier formation in cerebral microvessel endothelial cells. Am J Physiol Regul Integr Comp Physiol.

[39] Anna Z, Angela S, Barbara B, Jana R, Tamara B, Csilla V (2014). Heart-protective effect of n-3 PUFA demonstrated in a rat model of diabetic cardiomyopathy. Mol Cell Biochem.

[40] Nygren A, Olson ML, Chen KY, Emmett T, Kargacin G, Shimoni Y (2007). Propagation of the cardiac impulse in the diabetic rat heart: reduced conduction reserve. J Physiol.

[41] Sheu JJ, Chang LT, Chiang CH, Sun CK, Chang NK, Youssef AA (2007). Impact of diabetes on cardiomyocyte apoptosis and connexin43 gap junction integrity: role of pharmacological modulation. Int Heart J.

[42] Zhou D, Yang Y, Chen J, Zhou J, He J, Liu D (2024). N-acetylcysteine Protects Against Myocardial Ischemia-Reperfusion Injury Through Anti-ferroptosis in Type 1 Diabetic Mice. Cardiovasc Toxicol.

[43] Domínguez C, Ruiz E, Gussinye M, Carrascosa A (1998). Oxidative stress at onset and in early stages of type 1 diabetes in children and adolescents. Diabetes Care.

[44] Stockklauser-Färber K, Ballhausen T, Laufer A, Rösen P (2000). Influence of diabetes on cardiac nitric oxide synthase expression and activity. Biochim Biophys Acta.

[45] Kisacam MA, Kocamuftuoglu GO, Ufat H, Ozan ST (2022). The evaluation of early stage oxidative status in streptozotocin induced diabetes in rats. Arch Physiol Biochem.

[46] Mourmoura E, Vial G, Laillet B, Rigaudière JP, Hininger-Favier I, Dubouchaud H (2013). Preserved endothelium-dependent dilatation of the coronary microvasculature at the early phase of diabetes mellitus despite the increased oxidative stress and depressed cardiac mechanical function ex vivo. Cardiovasc Diabetol.

[47] Katyare SS, Satav JG (2005). Effect of streptozotocin-induced diabetes on oxidative energy metabolism in rat kidney mitochondria. A comparative study of early and late effects. Diabetes Obes Metab.

[48] Gadicherla AK, Wang N, Bulic M, Agullo-Pascual E, Lissoni A, De Smet M (2017). Mitochondrial Cx43 hemichannels contribute to mitochondrial calcium entry and cell death in the heart. Basic Res Cardiol.

